# PR-Index: Using the *h*-Index and PageRank for Determining True Impact

**DOI:** 10.1371/journal.pone.0161755

**Published:** 2016-09-14

**Authors:** Chao Gao, Zhen Wang, Xianghua Li, Zili Zhang, Wei Zeng

**Affiliations:** 1 College of Computer and Information Science, Southwest University, Chongqing, China; 2 Interdisciplinary Graduate School of Engineering Sciences, Kyushu University, Fukuoka, 816-8580, Japan; 3 School of Information Technology, Deakin University, Locked Bag 20000, Geelong, VIC 3220, Australia; Yunnan University of Finance and Economics, CHINA

## Abstract

Several technical indicators have been proposed to assess the impact of authors and institutions. Here, we combine the *h*-index and the PageRank algorithm to do away with some of the individual limitations of these two indices. Most importantly, we aim to take into account value differences between citations-evaluating the citation sources by defining the *h*-index using the PageRank score rather than with citations. The resulting PR-index is then constructed by evaluating source popularity as well as the source publication authority. Extensive tests on available collections data (i.e., Microsoft Academic Search and benchmarks on the SIGKDD innovation award) show that the PR-index provides a more balanced impact measure than many existing indices. Due to its simplicity and similarity to the popular *h*-index, the PR-index may thus become a welcome addition to the technical indices already in use. Moreover, growth dynamics prior to the SIGKDD innovation award indicate that the PR-index might have notable predictive power.

## Introduction

The problem of objectively assessing the impact of an individual author has been the subject of intense research in bibliometrics as well as many other fields of research. While it may be relatively easy to distinguish a Nobel Prize winner from an average researcher, it is much more difficult to rank all authors. Yet, many have tried, using quantitative technical analysis of various indicators ranging from the number of publications and patents to the number of citations. Such ranking systems have found widespread use in funding agencies and tenure committees across the world to supplement objective and comprehensive assessment of each individual researcher’s impact. These indicators can also provide a fast glimpse into a field of research and aid in identifying experts or, at minimum, the most productive and well-known authors. However, technical indicators are also relatively easy to manipulate, and so care must be exercised; a thorough determination of impact should always include a human evaluation as well.

Numerous indicators have already been proposed. These can be roughly classified into two groups: statistical-based indicators and graph-based indicators. Statistical-based indicators typically depend on the sheer number of publications, patents, citations, or co-authors. Among these, the *h*-index [1] is probably the most famous and widely used. Graph-based indictors, on the other hand, explore the relationships within an academic network, such as a publication-citation network, an author citation network or a co-author network. Author impact can be assessed based on the structural properties of such academic networks in lieu of statistical-based indicators.

The *h*-index [1], on which this proposed PR-index is based, has several notable advantages and desirable properties. Because of its simplicity and intuitive value, the *h*-index is used widely in several academic ranking systems, including the Web of Knowledge [2] and the Microsoft Academic Search [3]. However, *h*-index rankings may also be misleading and manipulated. For example, self-citations [4] could increase a ranking, although originally it was claimed that is not an issue. In addition, the *h*-index treats all citations equally, so it does not take into account the quality of each citation. These disadvantages led to the development of a now-large number of variants of the *h*-index. Batista et al. [5] have taken field dependence into consideration, thus making it possible to quantify an author’s scientific contribution across different research fields. Schreiber [6] has proposed the index *h*_*s*_ to eliminate the negative effects of self-citations. Liu et al. [7] have introduced a modification of the *h*-index for multi-authored papers with contribution-based author name ranking. Zhang has proposed h’-index [8] and *e*-index [9], which both consider the whole set of citation information available for each author. A review of many different variants of the *h*-index was performed by Lutz Bornmann et al. [10], who reviewed and tested 37 different *h*-index variants for consistency and correlations between them.

The many proposed variants of the *h*-index are aimed specifically at mending some of its aforementioned deficiencies, but so far, few have explicitly taken the rationality of the citations into consideration. Sometimes, citations may not reflect an author’s or publication’s status accurately [11–14]. The original *h*-index may give a high score to an author who has published many highly cited reviews. This reflects the popularity of these publications, but not always reflects their authority in moving the field ahead.

Compared to statistical-based indicators, graph-based indicators consider the relationship between authors and their publications based on co-authorship and citation networks. PageRank, formulated by Brin and Page [15, 16] for assigning a rank to all Web pages, is one of the most famous graph-based indicators. In an academic network, an author will receive a high PageRank score if he or she is cited by (a co-author with) many other high-impact authors. For example, although two authors may have the same number of citations (or co-authors), they may receive different PageRank scores because the quality of the citations (or the co-authors) is considered as well. We briefly mention here two PageRank algorithms that are based on different networks, as follows:

1. Citation networks of authors: Ding [17] has proposed a weighted PageRank algorithm based on the citation network of authors. In her work, an author will receive a high rank score if that author is cited by many well-respected authors.
2. Collaboration networks of authors: Liu et al. [18] have proposed a PageRank algorithm by considering the frequency of co-authorships and the total number of co-authors on articles. Using this algorithm, highly co-authored and prolific authors will gain reputation. Yan et al. [19] have provided an alternative perspective for measuring author impact by applying a weighted PageRank algorithm that considers citation and co-authorship network topology.

Moreover, Fiala et al. [20] have proposed a modified PageRank algorithm that considers the relationship between both co-authorship and citation. Moreover, they also integrated PageRank with a time factor in a subsequent work [21].

Although the PageRank algorithm shows a great promise in academic rankings, it has some limitations:

1. PageRank based on author citation relationships may exaggerate an author’s research impact to a certain extent. For example, if a less prominent author has co-authored papers with a famous scholar and published three or four highly cited papers, that author will receive a high PageRank score.
2. PageRank based on co-authorship may also not properly reflect an author’s research impact. If an author’s PageRank score is high, it just means that he or she is widely co-authored. This indicator may reward authors for adding extra names or more famous names to the author list.

To overcome some of the limitations of both statistical and graphbased indicators, we propose a new “PR-index,” which is a combination of both. In brief, the PR-index is a variant of the *h*-index, which instead of simply considering citations to the papers by using the PageRank score of each paper within the citation network, which won’t increase the computational complexity. Obviously, this requires both constructing the citation network for publications and determining their PageRank, but otherwise it is as straightforward as determining the *h*-index. By replacing the citations with the PageRank score of each paper within the citation network, we obtain an index where both the popularity and the relevance of each particular author’s works are properly taken into account.

In the remainder of this paper, we first present a detailed account of our method in the section of PR-index. Then, we introduce the main results obtained with the PR-index and compare them with the results obtained with other indices in the section of Experiments and discuss their implications, as well as the predictive power of the PR-index in the section of PR-index Sequence. Final, we conclude our contribution in the section of Conclusion.

## PR-index

### Motivation of PR-index

**Fig 1:**
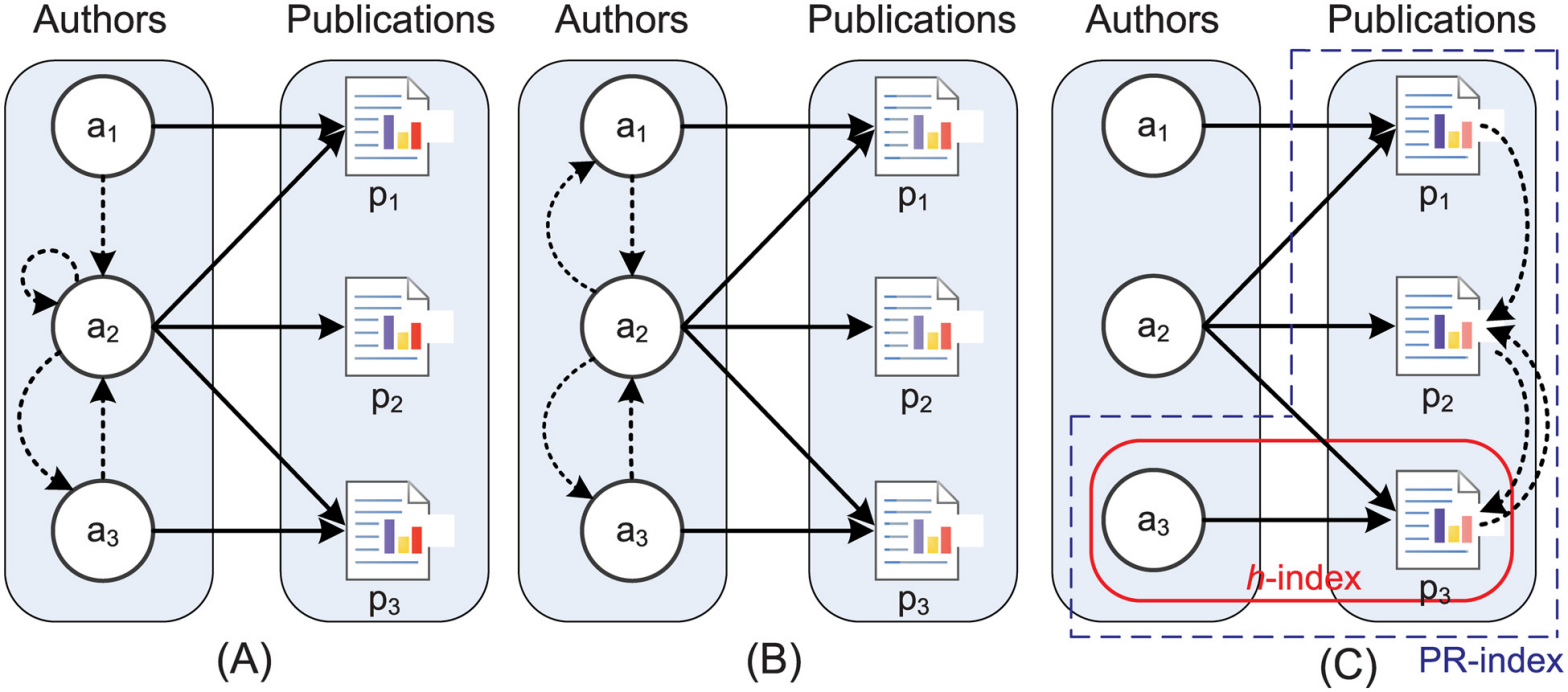
Three kinds of relationship in the academic research field. *a*1, *a*2, *a*3 denote three authors, *p*_1_, *p*_2_, *p*_3_ denote published papers. A black solid line means that an author published a paper. (a) illustrates the author citation relationship. The dashed arrow from *a*_1_ to *a*_2_ denotes that *a*_1_ cited *a*_2_ in a paper. (b) represents co-authorship among authors. There are two directed dashed edges between *a*_1_ and *a*_2_ which means that *a*_1_ has co-authored with *a*_2_. (c) represents for the publication citation relationship. A directed dashed edge from *p*_1_ to *p*_2_ means *p*_1_ has cited *p*_2_.

A bibliographic information network consists of rich information such as papers, authors and journals. As shown in Fig 1, there are three types of networks co-exist: the citation network of authors (Fig 1(a)), the co-authorship network of authors (Fig 1(b)), and the citation network of publications (Fig 1(c)). Fig 1 represents these relationships as black dotted lines. Given these relationships, we can define the problem of author evaluation as: How can we assess an author’s contribution according to these relationships?

The *h*-index, as a statistical-based indicator, was suggested by Hirsch [1] as a tool to determine authors’ impact. In Hirsch’s work, the index *h* for a scientist means that at least *h* papers from all his/her own *N*_*p*_ papers have been cited more than *h* times, and the other (*N*_*p*_ − *h*) papers have been cited fewer than *h* times. Actually, the information taken into consideration by the *h*-index is the red line area in Fig 1(c). In Fig 1(c), the *h*-index of *a*_3_ is 1 because he has published one paper that has been cited one time. Moreover, the *h*-index treats all citations equally and does not take the citation quality into consideration.

In addition to the *h*-index, which takes only statistical information into consideration, PageRank is also applied to the author impact assessment. Some authors [17–21] have modified its basic formula and applied it to both author and publication impact assessment. These methods can be grouped as follows:

1. PageRank based on author citation relationship (denoted as PR_AC). As shown in Fig 1(a), when an author is cited by many high-impact authors, he will achieve a high rank. In Fig 1(a), author *a*_2_ gets the first rank.
2. PageRank based on co-authorship (denoted as PR_CO). As shown in Fig 1(b), the more frequently the author collaborates with high-impact authors, the higher the rank that author will have. Author *a*_2_ gets the highest rank using this method as well.
3. PageRank based on publication citation relationship (denoted as PR_PC). As shown in Fig 1(c), when a paper is cited by many high-impact articles, that paper will receive a higher rank, such as the paper *p*_2_.

To overcome the limitations of the *h*-index and PageRank as discussed in the Introduction of this paper, the PR-index extends the *h*-index by combining it with PageRank. As shown in Fig 1, the PR-index considers publication and citation quantity but also takes a publication’s citation network into consideration. This means that the PR-index will rank majority authors higher by applying the PR_PC to distinguish high quality citations from low quality one.

### Formulation of PR-index

The main idea behind the PR-index is to calculate an *h*-index based on publication’s PageRank score rather than on citations. In some cases, a highly cited publication may not be of high quality. However, the PageRank score of such papers is much more reasonable because it takes both the popularity and the authority of each paper into consideration. We argue that an author who has a high *h*-index should have published many high-quality publications rather than many highly cited publications.

The process to create the PR-index consists mainly of calculating PageRank score, transforming PageRank score, and calculating the *h*-index.

**(1) PageRank score calculation**

First, we need to determine the PageRank score (PR_PC) for each paper. In the citation network of publications, the score of each paper can be worked out according to the following formula:

$$\begin{array}{c} PR\_PC\left(p\right)=\frac{1-d}{N}+d\sum_{i=1}^{k}\frac{PR\_PC(p_{i})}{Cite(p_{i})} \end{array}$$ (1)

where *N* represents the total number of papers, *p* is one paper and *p*_*i*_ is a paper that cites *p*. *PR*_*PC*(*p*) and *PR*_*PC*(*p*_*i*_) are the PageRank scores of paper *p* and *p*_*i*_, respectively; *Cite*(*p*_*i*_) is the sum of publications that cite *p*_*i*_.

**(2) PageRank score transformation**

First, we obtain a rank queue {*p*_1_, *p*_2_, …, *p*_*n*_} by sorting the *PR*_*PC* score for each paper in descending order.

Second, we need to calculate the *PRCite* score of paper *p*_*h*_ as follows:

$$\begin{array}{c} PRCite\left(p_{h}\right)=\frac{PR\_PC(p_{1})}{PR\_PC(p_{h})}Cite\left(p_{1}\right) \end{array}$$ (2)

where *p*_1_ is the first rank paper. *PR*_*PC*(*p*_1_) and *PR*_*PC*(*p*_*h*_) are the corresponding PageRank scores of paper *p*_1_ and *p*_*h*_ respectively. *Cite*(*p*_1_) is the citation score for paper *p*_1_. The *PRCite* score of paper *p*_1_ is equal to *Cite*(*p*_1_).

Third, we revise the *PRCite* score so that the *PRCite* score of the last ranked paper is 0. The revised formula is:

$$\begin{array}{c} PRCite^{{}^{\prime }}\left(p_{h}\right)=PRCite\left(p_{h}\right)-PRCite\left(p_{n}\right) \end{array}$$ (3)

where *PRCite*(*p*_*n*_) is the score of the latest ranked paper in the first step.

Finally, for some papers, *PRCite*′ (*p*_*h*_) is greater than their citations, so we need to revise *PRCite*′ (*p*_*h*_) for all publications according to the formula below:

$$\begin{array}{c} CitationPR\left(p_{h}\right)=\left\{\begin{array}{c} PRCite^{\prime }\left(p_{h}\right),\;\;if\;PRCite^{\prime }\left(p_{h}\right)\leq Cite\left(p_{h}\right)\\ Cite(p_{h}),\;\;elsewise \end{array}\right. \end{array}$$ (4)

**(3) *h*-index calculation**

This step calculates each author’s *h*-index based on both the number of publications and *CitationPR*, resulting in a new modified *h*-index we have named “PR-index.”

The algorithm of PR-index can be briefed in Table 1.

**Table 1:** The algorithm of PR-index.

Algorithm 1 PR-index
Input: N, the publication citation network; M, the author publication matrix.
Output: PR-index
1. Calculating the PR_PC according to Eq (1)
2. Sorting PR_PC in descend order
3. Calculating PRCite according to Eq (2)
4. Revising PRCite according to Eq (3)
5. Revising PRCite again according to Eq (4)
6. Calculating each author’s *h*-index based on Eq (5)
7. Outputting PR-index

## Experiments

### Dataset

By Microsoft Academic Search API [22], we extracted publicly available data from Microsoft Academic Search [3] based on the keyword of “*Data Mining*” from 1992 to 2011. This dataset contains publication information, including title, authors, publication references, and so on. The dataset contains a total of 32410 publications and 51938 authors.

### The Distribution of Each Indicator

**Fig 2:**
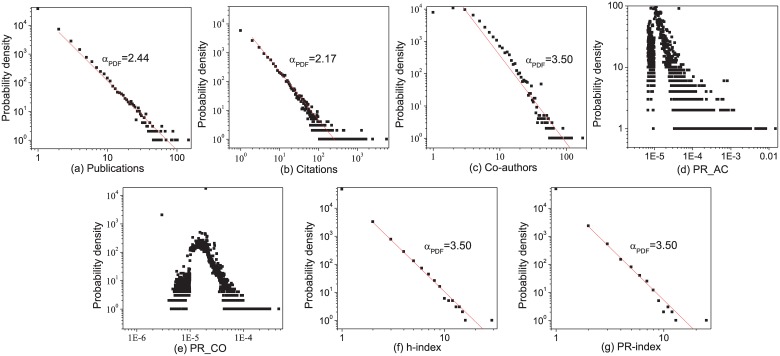
The distribution of different indicators with logarithmic scales. The x-axes denote different indicators, i.e., the total number (a) publications and (b) citations, the total number of (c) cooperations with others, the values of (d) PR_AC and (e) PR_Co based on the author citation network and co-authorship network respectively, and the values of (f) *h*-index and (g) PR-index. From this figure, some indicators (i.e., Publications, Citations, Co-authors, *h*-index and PR-index) approximately follow a power law distribution. The power-law exponents are estimated with the maximum likelihood estimation based on the Matlab toolkit provided by Newman [23]. While, the log-log plots of PageRank as shown in (d, e) do not follow a power-law distribution.

Based on the data collected from Microsoft Academic Search [3], the indicators introduced and described in the Method Section (i.e., the number of publications, citations and co-authors, as well as PR_AC, PR_CO, the *h*-index and PR-index), can be determined and evaluated for consistency and relevance. Fig 2 plots the distribution of different indicators with logarithmic charts. The number of publications, citations, co-authors, the *h*-index and the PR-index all exhibit a fat tail and may be approximated by a power law.

### Correlation Analysis

**Fig 3:**
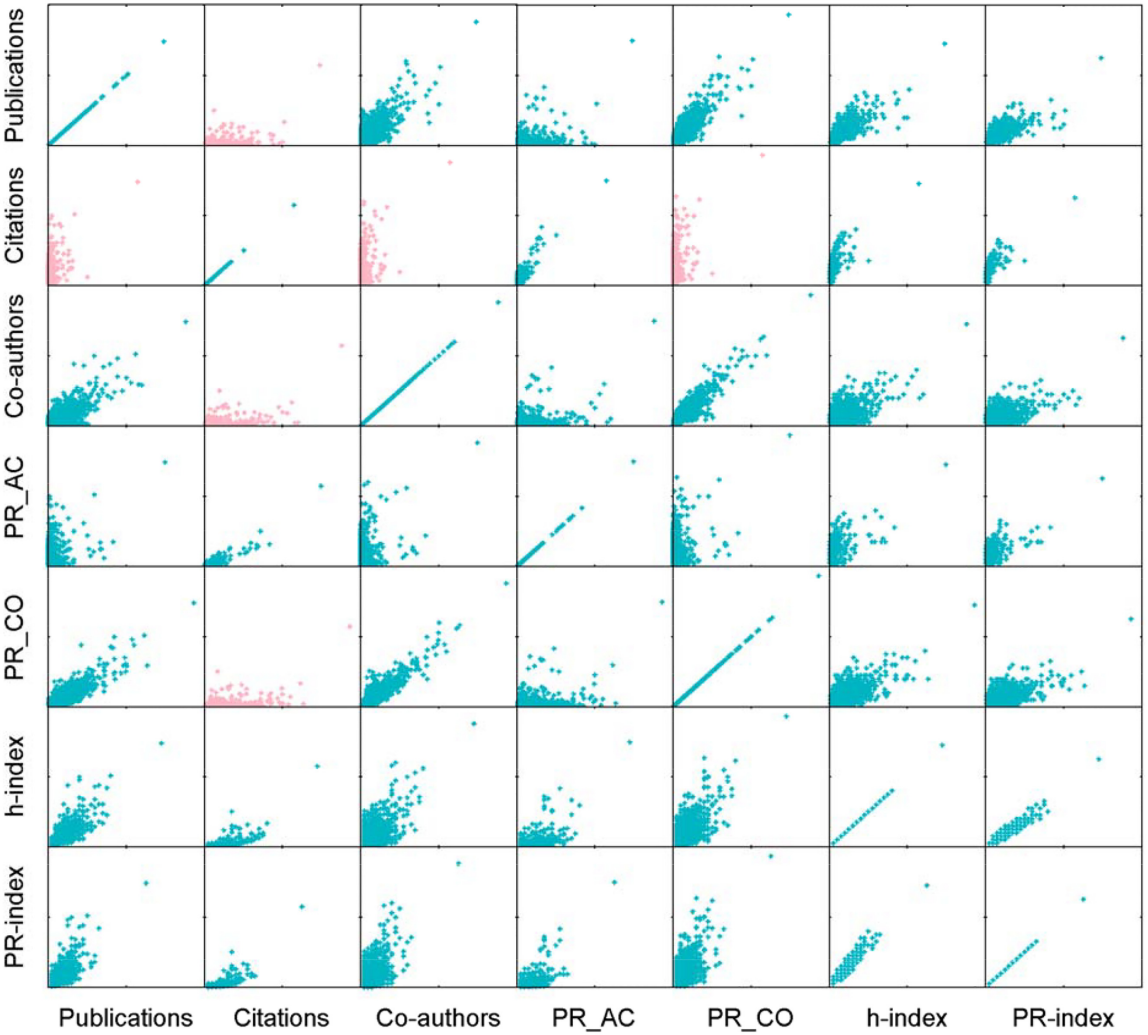
Illustration about the correlation among indicators based on the authors ranking of different indicators. The blue center dots in the principal diagonal show that two indicators highly correlate with each other. The sub figures in pink mean that two indicators are in a lower correlation obviously. This figure illustrates that the evaluation for the same author has the significant differences based on the indicators of Publications and Citations.

The scatter diagrams in Fig 3 give an intuitive analysis of the correlation relationship between each indicator. According to Fig 3, all indicators can be grouped into four groups according to their features: (1) publications; (2) co-authors, PR_CO; (3) citations, PR_AC; (4) *h*-index, PR-index.

Fig 3 shows that there is no significant correlation between publications and citations. This is because an author who has published many papers may still have few citations due to low-quality papers. Meanwhile, an author with many citations may have published only a few articles. This is the main reason why publications or citations alone cannot reflect an author’s achievement appropriately.

Co-authors and PR_CO are indicators based on cooperative relationships between authors. As shown in Fig 3, these two indicators have low correlation with other indicators, such as publications and citations. Actually, an author with high output and citations may receive a low ranking based on co-authors or PR_CO.

From the scatter diagrams, *h*-index and PR-index correlate well with other indicators. An author trying to achieve a higher rank must produce many high-quality papers to gain a better reputation.

## Discussion

### Comparison of Different Indicators

This section estimates which indicators objectively reflect authors’ impact in the field of scientific research. In bibliometrics, there are no standard indicators for reference. Sidiropoulos et al. [24] and Yan et al. [19] have evaluated award winners’ ranking results, reasoning that authors who have won awards should have a higher rank. This work adopts their method and uses the SIGKDD innovation award to evaluate the results of each indicator.

In Table 2, the top 20 authors given by different indicators are listed, with the SIGKDD innovation award winners shown in bold text. Clearly, JW Han is the most influential author, and receives the first rank in all indicators. Other authors, such as R Agrawal, UM Fayyad also achieve high ranks. Meanwhile, authors such as JH Friedman don’t place in the top 20.

**Table 2:** Each column presents the top 20 authors ranked by different indicators. Some authors who are SIGKDD by innovation award winners are highlighted with the boldface. The bracketed number indicates the scores of authors according to different indicators.

Publications	Citations	Co-authors	PR_AC	PR_CO	h-index	PR-index
**JW Han(148)**	**JW Han(5736)**	**JW Han(176)**	**JW Han(0.01494)**	**JW Han(4.66E-04)**	**JW Han(29)**	**JW Han(25)**
PS Yu(102)	M Kamber(2510)	RL Grossman(120)	**R Agrawal(0.00834)**	Q Yang(3.17E-04)	MJ Zaki(16)	MJ Zaki(13)
TP Hong(100)	PS Yu(1694)	Q Yang(116)	M Kamber(0.00718)	PS Yu(3.07E-04)	C Clifton(15)	SJ Stolfo(12)
Y Shi(96)	**R Agrawal(1574)**	PS Yu(112)	**P Smyth(0.00686)**	AA Freitas(2.75E-04)	PS Yu(15)	C Clifton(12)
AA Freitas(88)	IH Witten(1526)	**H Mannila(106)**	IH Witten(0.00667)	Y Shi(2.69E-04)	SJ Stolfo(14)	**R Agrawal(11)**
S Tsumoto(88)	**P Smyth(1428)**	**V Kumar(100)**	**UM Fayyad(0.00666)**	RL Grossman(2.52E-04)	**UM Fayyad(14)**	**UM Fayyad(11)**
A Kusiak(86)	J Pei(1331)	J Pei(95)	PS Yu(0.00593)	**H Mannila(2.50E-04)**	B Liu(14)	J Srivastava(11)
MJ Zaki(84)	MS Chen(1304)	Y Shi(89)	E Frank(0.00581)	J Pei(2.48E-04)	EJ Keogh(13)	PS Yu(10)
XD Wu(70)	**UM Fayyad(1304)**	XD Wu(89)	**R Srikant(0.00543)**	XD Wu(2.46E-04)	**H Mannila(13)**	OR Zaiane(10)
**V Kumar(68)**	E Frank(1275)	B Liu(85)	G Piatetsky-shapiro(0.00523)	**C Faloutsos(2.40E-04)**	J Srivastava(13)	**V Kumar(9)**
CQ Zhang(68)	Y Yin(1192)	W Wang(81)	MS Chen(0.00503)	B Liu(2.34E-04)	OR Zaiane(12)	R Kohavi(9)
**H Mannila(61)**	**R Srikant(1030)**	AA Freitas(79)	**H Mannila(0.00455)**	W Wang(2.14E-04)	AA Freitas(12)	B Liu(9)
EJ Keogh(61)	C Clifton(994)	H Blockeel(76)	JS Park(0.00335)	YL Chen(2.13E-04)	H Kargupta(12)	AA Freitas(9)
VS Tseng(61)	**V Kumar(972)**	C Faloutsos(74)	J Pei(0.00328)	**V Kumar(2.13E-04)**	**R Agrawal(12)**	EJ Keogh(9)
S Parthasarathy(60)	G Piatetsky-shapiro(935)	MJ Zaki(71)	M Mehta(0.00326)	TP Hong(2.12E-04)	**V Kumar(12)**	**H Mannila(8)**
**C Faloutsos(59)**	J Srivastava(928)	OR Zaiane(70)	Y Yin(0.00315)	OR Zaiane(2.10E-04)	H Toivonen(11)	M Chen(8)
Q Yang(59)	**H Mannila(913)**	HP Kriegel(69)	N Cercone(0.00283)	MJ Zaki(2.07E-04)	A Kusiak(11)	K Shim(8)
RL Grossman(58)	MJ Zaki(823)	N Ramakrishnan(66)	J Rissanen(0.00270)	A Kusiak(2.00E-04)	R Kohavi(11)	R Rastogi(8)
H Kargupta(56)	PN Tan(784)	H Chen(64)	SJ Stolfo(0.00266)	VS Tseng(1.89E-04)	W Lee(11)	W Lee(8)
Jerzy W. Grzymala-Busse (54)	SJ Stolfo(775)	C Clifton(64)	J Shafer(0.00265)	T Menzies(1.83E-04)	W Hsu(11)	H Toivonen(8)

The ranks of the SIGKDD innovation award winners are presented in Table 3, which shows that citations, PR_AC, *h*-index and PR-index result in a higher rank for these winners. In contrast, their publications, PR_CO and co-authors rankings are quite low. The following paragraphes discuss the ranking of each indicator in more depth.

**Table 3:** Comparison of ranks of authors who are SIGKDD innovation award winners based on different indicators. The boldface refers to the minimum number of each row. PR_AC gives the highest ranking to awarded authors. Leo Breiman who awarded the SIGKDD innovation award in 2005 is not list in this table. Actually, our dataset is clawed based on the keyword “*data mining*” and Prof. Leo mainly focuses on the statistics and machine learning. Therefore, there are a few records of Leo Breiman in our dataset.

Awarded Authors	Publications	Citations	Co-authors	PR_AC	PR_CO	h-index	PR-index
JW Han	**1**	**1**	**1**	**1**	**1**	**1**	**1**
UM Fayyad	68	9	440	6	258	**5**	**5**
H Mannila	13	17	**5**	12	7	8	16
V Kumar	10	14	**6**	23	14	11	10
R Agrawal	64	4	70	**2**	79	11	5
P Smyth	138	6	499	**4**	281	37	23
R Ramakrishnan	108	52	442	59	361	37	**23**
C Faloutsos	16	59	14	53	**10**	37	23
R Srikant	375	12	2042	**9**	2023	60	41
JH Friedman	714	33	8545	**27**	1633	94	79
Averange Rank	150.7	20.7	1206.4	**19.6**	466.7	30.1	22.6

(1) ***Publications***

It is well known that ranking authors by the total number of publications has some shortcomings. Such a ranking places lopsided emphasis on authors’ output while omitting consideration of the quality of their papers. In Table 3, the rank given by the total number of publications is quite low, which also reflects its unsuitability as an indicator.

(2) ***Co-authods and PR_CO***

Co-authors and PR_CO tend to provide lower author rankings compared to other indicators. As Table 3 shows, some award-winning authors receive a low rank because they have not co-authored with many other authors. However, in reality, these authors have published many high-impact articles.

Currently, PR_CO is recognized as a useful indicator in the area of informetrics [19]. In this work, we argue that PR_CO simply reflects the centrality of an author in a co-author network. By co-authoring with numerous authors (such as adding many authors who have contributed nothing in the author list of a paper), an author can increase their ranking even if most of those co-authors have average rankings. Thus, sometimes, having large numbers of co-authors is not meaningful.

(3) ***Citations and PR_AC***

As Table 3 shows, citations and PR_AC are the indicators which give the highest rank to the award winners. Ding’s work has shown that PR_AC is a well-designed indicator and concludes that PR_AC is better than other indicators [17].

However, when we focused on the essence of these two indicators in the original dataset, we found that some authors have an inappropriate rank. Table 4 lists the rank of the top 20 authors as measured by PR_AC along with their scores on other indicators are both presented. The items in bold text are authors who have an inappropriate rank. For example, Y Yin, who has published 7 papers, with 1192 citations and 7 co-authors gets a rank of 16. That’s because Y Yin co-authored with JW Han and published the paper *Mining frequent patterns without candidate generation*, which has acquired 840 citations. Meanwhile, Yin is the third author of this paper. This paper’s high citation count leads directly to Yin’s high ranking. So, if PR_AC or citations serve as standard indicators, they may cause a misleading result.

**Table 4:** Comparison of ranks of the top 20 authors based on the indicator of PR_AC. The authors who are in the boldface may get an inappropriate ranking for they only published a few papers. Numerical in the brackets stands for authors’ rank according to different indicators. Due to the limitation of table width, there are some abbreviations in this table. The Pub. is short for Publications, Co. is short for Co-authors.

Rank	Name	Pub.	Citations	Co.	PR_AC	PR_CO	*h*-index	PR-index
1	JW Han	148(1)	5736(1)	176(1)	0.014943(1)	0.000466(1)	28(1)	24(1)
2	R Agrawal	35(64)	1574(4)	44(70)	0.008336(2)	0.000119(79)	11(14)	11(3)
3	M Kamber	12(524)	2510(2)	19(796)	0.007183(3)	0.000042(1408)	7(60)	6(41)
4	P Smyth	25(135)	1428(6)	23(499)	0.006857(4)	0.000075(281)	8(37)	7(23)
5	IH Witten	25(135)	1526(5)	27(311)	0.006671(5)	0.000084(206)	7(61)	7(24)
6	UM Fayyad	34(68)	1304(8)	24(441)	0.006663(6)	0.000077(258)	13(5)	11(4)
7	PS Yu	102(2)	1694(3)	112(4)	0.005928(7)	0.000307(3)	15(2)	10(8)
8	E Frank	21(205)	1275(10)	17(1016)	0.005807(8)	0.000056(653)	7(62)	7(25)
9	R Srikant	15(373)	1030(12)	13(2040)	0.005427(9)	0.000037(2023)	7(63)	6(42)
10	G Piatetsky-shapiro	25(135)	935(15)	41(124)	0.005225(10)	0.000112(94)	8(38)	6(43)
11	MS Chen	44(36)	1304(9)	30(222)	0.005030(11)	0.000135(49)	9(25)	8(14)
12	H Mannila	61(12)	913(17)	106(5)	0.004545(12)	0.00025(7)	13(6)	8(15)
13	JS Park	10(712)	760(21)	5(11252)	0.003348(13)	0.000021(9623)	6(94)	6(44)
14	J Pei	50(25)	1331(7)	95(7)	0.003281(14)	0.000248(8)	9(26)	7(26)
**15**	**M Mehta**	**5(2170)**	**492(35)**	**6(8545)**	**0.003262(15)**	**0.000018(31834)**	**4(249)**	**4(132)**
**16**	**Y Yin**	**7(1300)**	**1192(11)**	**7(6672)**	**0.003154(16)**	**0.000023(7057)**	**5(150)**	**5(79)**
17	N Cercone	25(135)	189(112)	27(311)	0.002832(17)	0.000079(240)	5(151)	4(133)
**18**	**J Rissanen**	**2(7151)**	**174(131)**	**2(31374)**	**0.002695(18)**	**0.000008(48362)**	**2(981)**	**2(525)**
19	SJ Stolfo	36(56)	775(20)	32(194)	0.002659(19)	0.000087(190)	13(7)	11(5)
**20**	**J Shafer**	**4(2933)**	**318(57)**	**5(11252)**	**0.002647(20)**	**0.000015(38319)**	**3(440)**	**3(239)**

(4) **h-*index and PR-index***

When compared with citations and PR_AC, *h*-index and PR-index both assign a lower rank to the award winners. This means that authors such as Y Yin will receive a lower rank (*h*-index rank is 150; PR-index rank is 79). In fact, an author will be ranked highly by *h*-index and PR-index only if that author has truly published many influential papers.

Compared with the *h*-index, the PR-index assigns a higher rank to awarded authors, because the PR-index is based on publication quality rather than citations. Earlier in this paper, we discussed a theoretical shortcoming of the *h*-index which may exaggerate the ranking of authors who have published many highly cited reviews. The results here are evidence that high numbers of citations don’t necessarily equal high quality work. The PR-index is based on the PageRank score of publications rather than on citations, which optimizes the ranking results to some degree.

### PR-index Sequence

In Liang’s view [25], the *h*-index does not address different evolution mechanism for each author. His paper proposes the h-sequence instead, and discusses the evolution mechanism. This section aims to explore the evolution mechanism of the PR-index for those authors ranked the highest by the PR-index.

**Fig 4:**
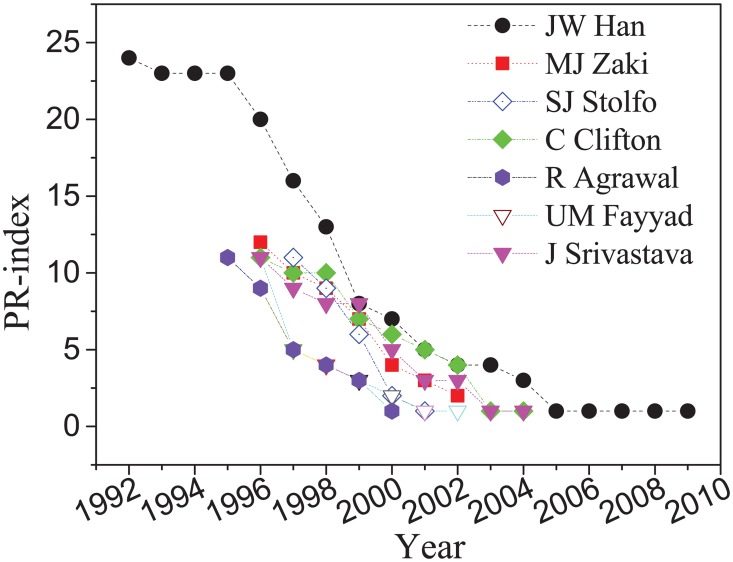
Illustration of evolution mechanism of PR-index sequence. The top of 7 authors are selected based on the ranking of PR-index in Table 2. The PR-index presents different types of decreasing patterns based on the both citations and publications.

Based on the ranking results of PR-index in Table 2, the top of 7 authors are selected as an example to illustrate the evolution mechanism of PR-index sequence. As shown in Fig 4, the PR-index indicator of each author decreases over time. In Hirsch’s work, it is expected that the *h*-index will decrease linearly. In contrast, Liang believes that there are different types of evolution mechanisms that can affect the decrease of *h*-index. Therefore, an decrease in *h*-index can be linear curve, “s” curve, and Lorenz curve. The PR-index also presents these dynamic types. Intuitively, JW Han is the most influential author, and has a PR-index of 25. Han has declined rapidly from 1995 to 1999, but makes slow progress from 2005 to 2011. Moreover, the evolution mechanism of PR-index for SIGKDD innovation award winners (i.e., UM Fayyad and R Agrawal) is different from other authors who have not won this award. Fayyad and Agrawal decreased rapidly from 1995 to 1996 due to their important fundamental contribution in their early research career.

Table 5 presents the PR-index sequences, which shows that Han’s research career is the longest. Most of these authors started their impact research in Data Mining from 1995 to 1997. To compare authors who have the same PR-index, we define their most productive 5 years (MP5), a measure that was introduced in Liang’s paper [25] as follows:

$$\begin{array}{c} {\text{MP5 }=\text{ PR}-\text{index}}_{\text{y}+4}-{\text{PR}-\text{index}}_{\text{y}} \end{array}$$ (5)

where *y* refers to the year and PR-index_y_ refers to the PR-index of an author in year *y*. Table 5 lists the *MP*5 for each author. Appropriately, JW Han gets the maximum score. Moreover, SJ Stolfo receives the maximum score among 5 authors with the same PR-index (i.e., PR-index = 11). It can be concluded that Stolfo’s efficiency is the greatest among these authors because he has the highest MP5.

**Table 5:** The PR-index sequences and *MP*5 of the top 7 authors based on the PR-index indicator.

	JW Han	MJ Zaki	C Clifton	SJ Stolfo	UM Fayyad	J Srivastava	R Agrawal
**2011**	-	-	-	-	-	-	-
**2010**	-	-	-	-	-	-	-
**2009**	1	-	-	-	-	-	-
**2008**	1	-	-	-	-	-	-
**2007**	1	-	-	-	-	-	-
**2006**	1	-	-	-	-	-	-
**2005**	1	-	-	-	-	-	-
**2004**	3	-	1	-	-	1	-
**2003**	4	-	1	-	-	**1**	-
**2002**	4	2	**4**	-	1	**3**	-
**2001**	5	3	**5**	**1**	1	**3**	-
**2000**	**7**	**4**	**6**	**2**	**2**	**5**	1
**1999**	**8**	**7**	**7**	**6**	**3**	**8**	**3**
**1998**	**13**	**9**	**10**	**9**	**4**	8	**4**
**1997**	**16**	**10**	10	**11**	**5**	9	**5**
**1996**	**20**	**12**	*11*	-	**11**	*11*	**9**
**1995**	**23**	-	-	-	-	-	**11**
**1994**	23	-	-	-	-	-	-
**1993**	23	-	-	-	-	-	-
**1992**	*24*	-	-	-	-	-	-
**MP5**	16	8	6	10	9	7	8

## Conclusions

In conclusion, this paper proposes a new variant of *h*-index, PR-index, and also discusses the features of such indicator. PR-index was developed based on both *h*-index and PageRank to evaluate an author’s impact from an objective point of view. The core idea of PR-index is that it replaces the *h*-index’s consideration of citation with the PageRank score. Using this modification, both the popularity and authority of each publication are considered. As has been shown in our experimental results, the PR-index is more reasonable than other author-impact assessment indicators when taking the SIGKDD innovation award as an evaluation criterion.

Moreover, we have used a sequence analysis of PR-index to explore the evolution mechanism for the top authors by adopting Liang’s method [25] and illustrating the PR-index sequence for each author. According to the statistical results, we found that the evolution mechanism of PR-index sequence is varied with authors. As for our future work, we will try to take a combination with PageRank and other variants of *h*-index to eliminate other shortcomings of h-index. What’s more, we will explore the impact evolution of each author or institution and predict the future impact of each one.
